# Structural variation and parallel evolution of apomixis in citrus during domestication and diversification

**DOI:** 10.1093/nsr/nwac114

**Published:** 2022-06-14

**Authors:** Nan Wang, Xietian Song, Junli Ye, Siqi Zhang, Zhen Cao, Chenqiao Zhu, Jianbing Hu, Yin Zhou, Yue Huang, Shuo Cao, Zhongjie Liu, Xiaomeng Wu, Lijun Chai, Wenwu Guo, Qiang Xu, Brandon S Gaut, Anna M G Koltunow, Yongfeng Zhou, Xiuxin Deng

**Affiliations:** Key Laboratory of Horticultural Plant Biology (Ministry of Education), Huazhong Agricultural University, Wuhan 430070, China; Key Laboratory of Horticultural Plant Biology (Ministry of Education), Huazhong Agricultural University, Wuhan 430070, China; Key Laboratory of Horticultural Plant Biology (Ministry of Education), Huazhong Agricultural University, Wuhan 430070, China; Key Laboratory of Horticultural Plant Biology (Ministry of Education), Huazhong Agricultural University, Wuhan 430070, China; Key Laboratory of Horticultural Plant Biology (Ministry of Education), Huazhong Agricultural University, Wuhan 430070, China; Key Laboratory of Horticultural Plant Biology (Ministry of Education), Huazhong Agricultural University, Wuhan 430070, China; Key Laboratory of Horticultural Plant Biology (Ministry of Education), Huazhong Agricultural University, Wuhan 430070, China; Key Laboratory of Horticultural Plant Biology (Ministry of Education), Huazhong Agricultural University, Wuhan 430070, China; Key Laboratory of Horticultural Plant Biology (Ministry of Education), Huazhong Agricultural University, Wuhan 430070, China; Shenzhen Branch, Guangdong Laboratory of Lingnan Modern Agriculture, Genome Analysis Laboratory of the Ministry of Agriculture and Rural Affairs, Agricultural Genomics Institute at Shenzhen, Chinese Academy of Agricultural Sciences, Shenzhen 518124, China; Shenzhen Branch, Guangdong Laboratory of Lingnan Modern Agriculture, Genome Analysis Laboratory of the Ministry of Agriculture and Rural Affairs, Agricultural Genomics Institute at Shenzhen, Chinese Academy of Agricultural Sciences, Shenzhen 518124, China; Key Laboratory of Horticultural Plant Biology (Ministry of Education), Huazhong Agricultural University, Wuhan 430070, China; Key Laboratory of Horticultural Plant Biology (Ministry of Education), Huazhong Agricultural University, Wuhan 430070, China; Key Laboratory of Horticultural Plant Biology (Ministry of Education), Huazhong Agricultural University, Wuhan 430070, China; Key Laboratory of Horticultural Plant Biology (Ministry of Education), Huazhong Agricultural University, Wuhan 430070, China; Department of Ecology and Evolutionary Biology, University of California Irvine, Irvine, CA 92697, USA; Queensland Alliance for Agriculture and Food Innovation, University of Queensland, Brisbane 4072, Australia; Shenzhen Branch, Guangdong Laboratory of Lingnan Modern Agriculture, Genome Analysis Laboratory of the Ministry of Agriculture and Rural Affairs, Agricultural Genomics Institute at Shenzhen, Chinese Academy of Agricultural Sciences, Shenzhen 518124, China; Key Laboratory of Horticultural Plant Biology (Ministry of Education), Huazhong Agricultural University, Wuhan 430070, China

**Keywords:** genomics, evolution, population genetics, apomixis

## Abstract

Apomixis, or asexual seed formation, is prevalent in *Citrinae* via a mechanism termed nucellar or adventitious embryony. Here, multiple embryos of a maternal genotype form directly from nucellar cells in the ovule and can outcompete the developing zygotic embryo as they utilize the sexually derived endosperm for growth. Whilst nucellar embryony enables the propagation of clonal plants of maternal genetic constitution, it is also a barrier to effective breeding through hybridization. To address the genetics and evolution of apomixis in *Citrinae*, a chromosome-level genome of the Hongkong kumquat (*Fortunella hindsii*) was assembled following a genome-wide variation map including structural variants (SVs) based on 234 *Citrinae* accessions. This map revealed that hybrid citrus cultivars shelter genome-wide deleterious mutations and SVs into heterozygous states free from recessive selection, which may explain the capability of nucellar embryony in most cultivars during *Citrinae* diversification. Analyses revealed that parallel evolution may explain the repeated origin of apomixis in different genera of *Citrinae*. Within *Fortunella*, we found that apomixis of some varieties originated via introgression. In apomictic *Fortunella*, the locus associated with apomixis contains the *FhRWP* gene, encoding an RWP-RK domain-containing protein previously shown to be required for nucellar embryogenesis in *Citrus*. We found the heterozygous SV in the *FhRWP* and *CitRWP* promoters from apomictic *Citrus* and *Fortunella*, due to either two or three miniature inverted transposon element (MITE) insertions. A transcription factor, *FhARID*, encoding an AT-rich interaction domain-containing protein binds to the MITEs in the promoter of apomictic varieties, which facilitates induction of nucellar embryogenesis. This study provides evolutionary genomic and molecular insights into apomixis in *Citrinae* and has potential ramifications for citrus breeding.

## INTRODUCTION

The term ‘citrus’ encompasses several species that are important horticultural fruit crops. Citrus fruit is produced in more than 140 temperate and tropical countries, with the annual harvest reaching 130 million tons [1]. Investigations suggest that citrus originated in the southeast foothills of the Himalayas [2]. The genus *Citrus* contains three principal species: mandarins (*Citrus reticulata*), pummelo (*C. grandis* or *C. maxima*) and citron (*C. medica*) [3]. Genetic evidence suggests that hybridization of these three species has led to additional cultivated species like sour orange (*C. aurantium*), sweet orange (*C. sinensis*), grapefruit (*C. paradisi*) and lemon (*C. limon*) [2–4]. Citrus species are included within *Citrinae*, a subtribe of the *Citroideae* subfamily within Rutaceae [5]. *Citrinae* also includes atalantia (*Atalantia buxifolia*), papeda (named Ichang papeda, *C. ichangensis*) [6], poncirus (named trifoliate orange, *Poncirus trifoliata*) [7] and kumquat (the wild Hongkong kumquat *Fortunella hindsii* and the domesticated kumquat *F. crassifolia*) [8].

Citrus cross-breeding has been hampered by the presence of apomixis, an asexual form of seed formation that can limit the recovery of sexual progeny [9]. Apomixis has been found in at least 78 families and 293 genera of flowering plants (https://uni-goettingen.de/en/423360.html) [10]. It is typically a dominant genetic trait, and several different mechanisms have independently evolved in angiosperms [11,12]. In well-studied apomict species, apomixis is facultative, in that the sexual process remains intact in the plant to some extent [13]. Poncirus (*Poncirus* genus), mandarins (*Citrus* genus) and most of the derived cultivars following hybridization in *Citrus*, including sour orange, sweet orange, grapefruit and lemon, can exhibit a form of apomixis termed nucellar embryony (polyembryony) [14]. *Fortunella* genus members, wild Hongkong kumquat and the domesticated kumquat, can display nucellar embryony while some varieties can be completely sexual (or monoembryony) [8].

In *Citrinae* exhibiting nucellar embryony, sexual reproduction is mechanistically functional, a type of facultative apomixis in which sexual and apomictic processes coexist [15]. Meiosis occurs during male and female gametophyte formation giving rise to functionally reduced reproductive cells. The nucellar embryo initial cells arise from the nucellus surrounding the meiotically formed female embryo sac [15]. Competency for cells to undergo a nucellar embryogenic pathway in some species is stimulated as early as during the mitotic events of female gametogenesis [16]. Double fertilization is required to induce both zygotic embryo formation and nutritive endosperm formation to nourish the developing nucellar embryo initial cells to maturity [17]. They appear to utilize the nutrients from the degrading nucellus to initiate early embryogenic events, however, they cannot develop into mature embryos without additional access to the endosperm provided from sexual double fertilization of a meiotically derived embryo sac [15]. Nucellar or adventitious embryony generates many maternally derived embryos in the mature seed, in addition to a sexually derived embryo, therefore, multiple embryos germinate from a polyembryonic seed [18]. Depending on the genus, variety and the underpinning genetic capacity to generate multiple nucellar embryos, the sexually derived embryo may or may not survive [19].

Nucellar embryony resembles a potential change in fate, from a nucellar cell in the ovule to an embryogenic pathway in the absence of fertilization [20]. By contrast, in diplosporous and aposporous apomicts such as *Taraxacum* and *Hieracium*, respectively, embryos form without fertilization (termed parthenogenesis) from mitotically derived eggs inside a mitotically derived embryo sac structure [21–23]. Independent loci control mitotic embryo sac formation and parthenogenesis in diplosporous *Taraxacum* (dandelion) [24] and aposporous *Hieracium* and *Pennisetum* [25,26]. Parthenogenesis in both *Taraxacum* and *Hieracium* (now termed *Pilosella*) is controlled by the same gene encoding a K2-2 zinc finger protein contained EAR domain where a miniature inverted repeat transposable element (MITE) insertion in the promoter enables egg cell expression, supporting the transition to embryogenesis [27]. Parthenogenesis in these two eudicot members of Asteraceae, *Taraxacum* and *Hieracium*, appears to be a case of parallel evolution [27]. In aposporous monocot *Pennisetum*, parthenogenesis is controlled by *PsASGR-BABY BOOM-like* (*PsASGR-BBML*) encoding an APETALA2 transcription factor protein family member [28]. This demonstrates the recruitment of different genes to induce the fertilization-independent embryogenic component of apomixis in the plant kingdom [29]. The consequence of apomixis is the formation of clonal offspring with a maternal genotype [30]. It is known that clonal propagation can hide deleterious variants in a heterozygous state, including structural variants (SVs) that escape from recessive selection (selection against recessive alleles) [31,32]. The difference is that sexual reproduction is maintained in apomicts, and retention of sexual reproduction at 5% can halt the accumulation of deleterious mutations [33]. This raises the question of apomixis and its effects on genetic load in citrus, particularly given that this mode of reproduction spans a large evolutionary time scale.

Early genetic studies on *Citrus* species identified a 380 kb region on chromosome 4 of the mandarin genome required for nucellar embryony [34]. This region was further narrowed to 80 kb in the pummelo genome and contained an RWP-RK domain gene called *CitRWP* [6]. *CitRWP* encodes an RWP-RK domain-containing protein similar to the *Arabidopsis* RWP-RK domain (RKD) family of proteins shown to function in the maintenance of egg-cell identity and they have been shown to actively promote embryogenesis when they are ectopically expressed [35]. The *CitRWP* gene contains a MITE insertion in its promoter and association of the MITE insertion in the *CitRWP* gene correlated well with most varieties tested and known to undergo nucellar embryony [6]. A knockdown of *CitRWP* resulted in loss of nucellar embryony in one successfully regenerated sweet orange, while sexual reproduction remained intact, suggesting it is required for nucellar embryo formation in sweet orange [36]. In addition, there is evidence that apomixis alleles are transmitted in modern mandarins via hybridization and introgression [37]. Despite these genetic advances, the evolution of apomixis within *Citrinae* has not been investigated broadly. It remains unclear to what extent the distribution of nucellar embryony across *Citrinae* is due to introgression via hybridization events, if it has independently evolved via the recruitment of different genes or by parallel evolution, and/or combinations of the latter.

In this paper, for addressing these questions, a high-quality chromosomal genome assembly of Hongkong kumquat (*F. hindsii*) was generated and 46 accessions of Hongkong kumquat were sequenced. We built a genome-wide variation map based on the whole genome resequencing (WGS) data of 234 accessions and 6 genomes from different *Citrinae* genera. Analyses were conducted to examine if the distribution of nucellar embryony across species was due to introgression or caused by parallel evolution. In addition, the population genomic analysis confirmed the sheltering of heterozygous deleterious variants in apomictic hybrid origin populations. Quantitative trait locus (QTL) mapping was used to identify genetic regions that contribute to nucellar embryony in *Fortunella* in comparison to those known in *Citrus*. The captured long-reads sequencing, which supported two or three MITE insertions in the *CitRWP* or *FhRWP* promoter, were required to initiate expression. Within the apomixis region, haplotype phylogenies revealed that the heterozygous MITE insertions of the *RWP* promoter are common in apomictic citrus populations. Further, we identified a transcription factor encoded by the *FhARID* gene, which interacts with the MITE element in the *FhRWP* promoter. The data suggest parallel evolution of nucellar embryony in *Citrinae* genera and the implications for citrus breeding are discussed.

## RESULTS

### Genome assembly and structural variation in citrus assemblies

**Figure 1.**
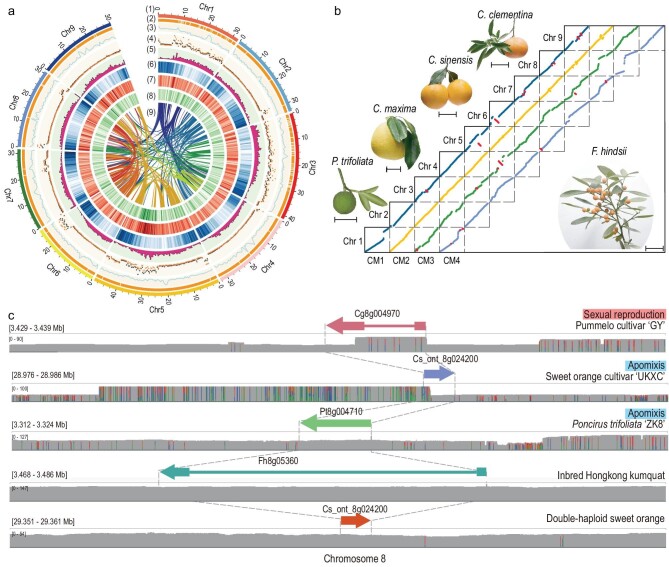
The Hongkong kumquat genome assembly and comparative genomics. (a) (1) Circular representation of the kumquat genome pseudochromosomes. (2) The order of the contigs in each chromosome. (3–6) The distribution of the GC density, heterozygosity density, gene density and repeat density, respectively, with densities calculated in 500 kb windows; points in (4) show the density of heterozygous small insertions and deletions (INDELs) and single nucleotide polymorphisms (SNPs), respectively. (7, 8) The presence/absence variation (PAV) density compared with the double-haploid sweet orange genome and the double-haploid pummelo genome. (9) Syntenic blocks showing the regions of nine citrus chromosomes sharing a common order of homologous genes. The band width is proportional to the syntenic block size. (b) Photos of five citrus varieties, Hongkong kumquat (*F. hindsii*), clementine (*C. clementina*), sweet orange (*C. sinensis*), pummelo (*C. maxima*) and poncirus (*P. trifoliata*). Scale bars, 5 cm. The putative inversion events in nine chromosomes are shown in the same color. The Hongkong kumquat genome (y axis) was compared with clementine (CM1), sweet orange (CM2), pummelo (CM3) and poncirus (CM4) genomes. (c) Integrative Genomics Viewer (IGV) plot showing the coverage of reads and the structure of a hemizygous gene in five assemblies with different reproductive systems. In each diagram, the arrow denotes the gene, and the vertical lines indicate the boundary of the coding sequence (CDS) region in the homologous genes. The bin color is indicated in the heterozygous sites in each window. The height represents the read coverage.

To facilitate the evolutionary, genetic and molecular analysis of nucellar embryony in *Citrinea*, an improved genome assembly of the Hongkong kumquat line Sy3-45 was generated. It had been previously assembled into contigs with a genome size of ∼389 Mb as estimated by K-mer distribution and flow cytometry [8]. The total length of the improved assembly was 323.72 Mb, with a contig N50 size of 9.77 Mb (Fig. 1a), which is a 4.4-fold improvement compared to the previous contig-level version (Table S1) [8]. Approximately 98 out of 107 (91.58%) contigs were anchored into 9 pseudochromosomes (Fig. S1 and Table S3). Further analysis of the pseudochromosome assembly revealed 97% complete and 2.5% duplicated Benchmarking Universal Single-Copy Orthologs (BUSCOs) (Table S2), suggesting the genome is fairly complete. Genome annotation revealed 32 563 gene models and also identified 46.11% of the genome as transposable elements (TEs) (Tables S4 and S5). We aligned and compared our improved kumquat assembly to the reference genomes of *Citrus clementina* [3], *C. sinensis* [38], *C. maxima* [6] and *P. trifoliata* [7]; our assembly was highly collinear with these *Citrinae* genomes (Fig. 1b).

To evaluate heterozygosity within genomes and the association of heterozygosity with the reproductive modes, we estimated genic hemizygosity based on presence and absence variants (PAVs) in five assemblies with deep long-read coverage (>100-fold). The five assemblies included: a doubled-haploid sweet orange, which served as homozygous control; our inbred Hongkong kumquat; a diploid pummelo cultivar, ‘GY’, that was sexually reproducing [39], an apomictic diploid sweet orange cultivar, ‘UKXC’ [38], and an apomictic diploid wild trifoliate orange, ‘ZK8’ (Fig. 1c and Table S6) [7]. We found the proportion of hemizygous genes (or gene structure missing >50%, *g_h_*) to be 2.0% in our improved kumquat assembly (Fig. 1c and Figs S2–S7). Among the five citrus assemblies, the apomictic sweet orange cultivar ‘UKXC’ had the highest *g_h_* estimate (11.2%), with the lowest *g_h_* estimate (0.06%) found in the doubled-haploid sweet orange, which might be an assembly error, because the doubled haploid should be fully homozygous, with a *g_h__ _= *0.00. Compared to other crops, *g_h_* of 11.2% is slightly lower in apomictic sweet orange than clonal grapevine (∼15.5%) [32], but higher than a perennial wild outcrossing rice (*Oryza longistaminata*) (∼9.0%) and domesticated selfing rice (0.35%–0.73%) [40]. Overall, these *g_h_* estimates add to the growing observation that genic hemizygosity is related to reproductive modes, with *g_h_* being higher in clonal and apomictic crops but lower in selfers.

### Multiple origins of apomixis in *Citrinae*

To further aid investigation into the evolution of nucellar embryony in *Poncirus*, *Fortunella* and *Citrus* of *Citrinae*, whole genome sequencing data were collected from 234 accessions throughout *Citrinae* (average coverage 36.24 ± 7.02-fold) [2,6,41,42], including 46 that were newly sequenced from Hongkong kumquat accessions (average coverage 37.55 ± 2.06-fold) (Fig. S8 and Table S8). The WGS data included sequences from taxa that are mixtures (i.e. grapefruit, sweet orange, lemon and sour orange) between apomictic mandarin and sexually reproducing pummelo and citron. After mapping resequencing data to our reference, we filtered low-quality variants and identified ∼6.9 million reliable SNPs among all samples. We performed principal component analysis (PCA) based on the data set with linkage disequilibrium (LD) pruning (∼1.4 million SNPs, see Methods) (Fig. 2c) and estimated a covariance matrix (Figs S10 and S12), for which PC1 (22.9%) and PC2 (19.2%) characterized different genera (Fig. 2c and Fig. S9) with a mixed apomixis phenotype. Given that some of these mixed taxa have a mandarin genomic component, their grouping suggested that hybridization has contributed to the distribution of the apomixis phenotype within *Citrus.* We also conducted STRUCTURE analyses with *K *= 2 to 12 (Fig. S10). A total of 12 populations were separated at an optimal grouping of *K *= 10 (Fig. 2a and Fig. S11), with clear genetic ancestry of citron, pummelo and mandarin and an obvious history of modern admixture in sweet orange, sour orange, lemon and grapefruit (Fig. 2a and Fig. S12).

**Figure 2.**
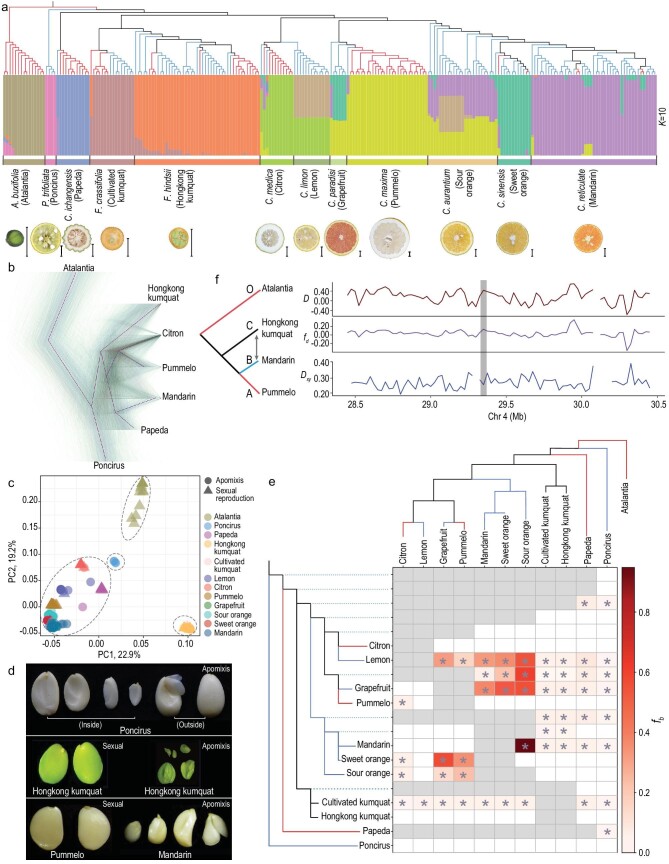
Phylogenetic and population structure analyses. (a) A maximum-likelihood phylogenic tree of 234 accessions using whole-genome SNP data. Branch colors denote the individuals with different reproductive systems. Model-based clustering analyses were carried out under the assumption of 10 ancestral clusters (*K* = 10) with some mixed populations. Species names, common names and mature fruits are indicated at the bottom. Scale bars, 1 cm. (b) A total of 3316 trees, and resulting consensus tree, of seven species were inferred in 50 kb non-overlapping windows. (c) PCA of 234 accessions. PC1 and PC2 are displayed. The four genera—*Atalantia*, *Poncirus*, *Fortunella* and *Citrus*—are circled. (d) Seeds from apomictic trifoliate orange (*P. trifoliata*), sexually reproductive and apomictic Hongkong kumquat (*F. hindsii*), sexual pummelo (*C. maxima*) and apomictic mandarin (*C. reticulata*). (e) The branch-specific statistic *f_b_* identified excess sharing of derived alleles between the different branches of the tree. The whole-genome maximum-likelihood phylogenetic tree was used as a basis for the branch statistic. Asterisks denote block jackknifing significance (Benjamini-Hochberg, *P* < 0.001). *Atalantia* was used as the outgroup in all comparisons. (f) Diagram of the phylogeny used to test for introgression between Hongkong kumquat and mandarin (left). *D* statistic, *f_d_* statistic and *D_xy_* of chromosome 4 were plotted (right) with the bar showing the candidate locus underlying the transition from sexual reproduction to apomixis in *Citrus*.

**Figure 2.**
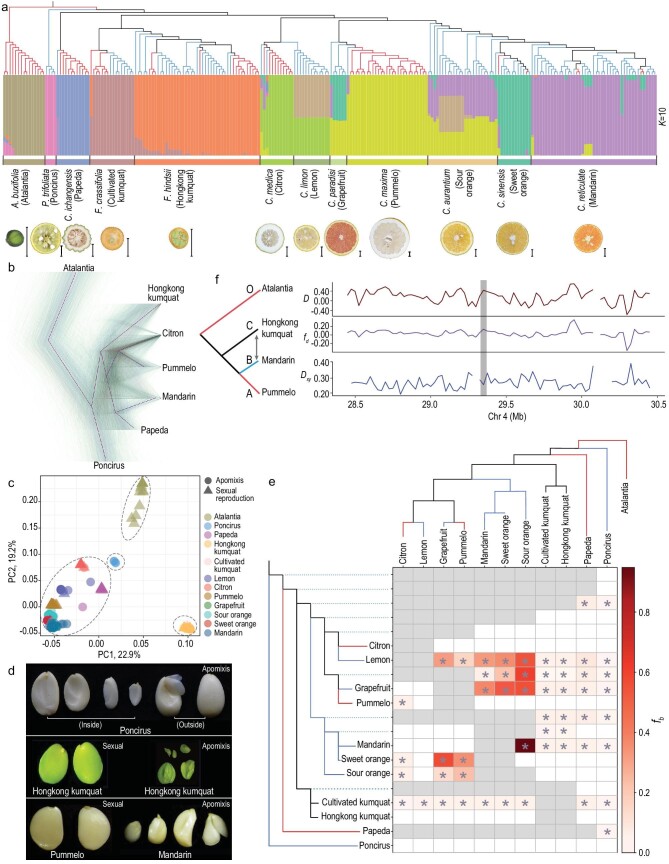
Phylogenetic and population structure analyses. (a) A maximum-likelihood phylogenic tree of 234 accessions using whole-genome SNP data. Branch colors denote the individuals with different reproductive systems. Model-based clustering analyses were carried out under the assumption of 10 ancestral clusters (*K* = 10) with some mixed populations. Species names, common names and mature fruits are indicated at the bottom. Scale bars, 1 cm. (b) A total of 3316 trees, and resulting consensus tree, of seven species were inferred in 50 kb non-overlapping windows. (c) PCA of 234 accessions. PC1 and PC2 are displayed. The four genera—*Atalantia*, *Poncirus*, *Fortunella* and *Citrus*—are circled. (d) Seeds from apomictic trifoliate orange (*P. trifoliata*), sexually reproductive and apomictic Hongkong kumquat (*F. hindsii*), sexual pummelo (*C. maxima*) and apomictic mandarin (*C. reticulata*). (e) The branch-specific statistic *f_b_* identified excess sharing of derived alleles between the different branches of the tree. The whole-genome maximum-likelihood phylogenetic tree was used as a basis for the branch statistic. Asterisks denote block jackknifing significance (Benjamini-Hochberg, *P* < 0.001). *Atalantia* was used as the outgroup in all comparisons. (f) Diagram of the phylogeny used to test for introgression between Hongkong kumquat and mandarin (left). *D* statistic, *f_d_* statistic and *D_xy_* of chromosome 4 were plotted (right) with the bar showing the candidate locus underlying the transition from sexual reproduction to apomixis in *Citrus*.

We also inferred a phylogenetic tree based on the ∼1.4 million LD pruning SNP data set (Fig. 2a; see Methods). The results separated *Atalantia*, *Poncirus*, papeda, kumquat (cultivated kumquat and Hongkong kumquat of *Fortunella*) and true citrus (including admixed cultivars), corroborating the PCA and STRUCTURE results. The phylogeny analysis suggested that the reproductive mode shifted on multiple occasions from complete sexual reproduction to apomictic and back to sexual (Fig. 2a). Based on the phenotype of reproductive modes, we elucidated three features of apomixis in *Citrinae*: (i) *Poncirus* is entirely apomictic, with the caveat that few individuals were sampled (*n* = 4); (ii) wild Hongkong kumquat and cultivated kumquat contain both apomictic and sexually reproducing individuals that were distinguishable by seed morphology (Fig. 2d); and (iii) most mandarin accessions were apomictic, as were accessions that contained genomic proportions from mandarin. However, some historical mandarin varieties are sexually reproductive, such as ‘Kyomi’ tangor (*Citrus unshiu* × *C. sinensis*) [43] and ‘Orha’ mandarin [‘Temple’ (Citrus temple hort. ex Y. Tanaka) × ‘Dancy’ (Citrus tangerine hort. ex Tanaka)] [44] (Table S8). Overall, the phylogeny demonstrates that the apomixis phenotype is widely distributed among *Citrinae*, and the result further suggests the possibility that apomixis may have had multiple origins in the group.

Important questions are whether apomixis has evolved in parallel in clades of *Citrinae* and to what extent nucellar embryony owes its origin to introgression across taxa. To distinguish introgression events from other processes such as incomplete lineage sorting (ILS) [45], which potentially produce similar genetic signatures, first, the genome-wide potential introgression was analyzed based on 12 populations, as defined by use of STRUCTURE, that assigned *Atalantia* as an outgroup based on the ∼6.9-million-SNPs data set (see Methods). The DensiTree was constructed using 50 kb non-overlapping windows and the result indicated some topological uncertainty that could be caused by introgression or ILS among *Citrinae* taxa (Fig. 2b and Fig. S13). The *f_b_* statistic is expected to be linear in relation to the genome-wide introgressed proportion [46]. Therefore, we also applied the *f_b_* statistic to detect introgression and estimate shared genomic variation. These analyses suggested that *Poncirus* (*f_b_* = 0.0352 ± 0.0155, *P* < 0.001, false discovery rate (FDR)), papeda (*f_b_* = 0.0654 ± 0.0298, *P* < 0.001, FDR) and Hongkong kumquat (*f_b_* = 0.0300 ± 0.0117, *P* < 0.001, FDR) significantly contributed to the gene pools of modern cultivars in *Citrus* (Fig. 2e). This finding was also supported by other frequency-based statistics i.e. *D* statistics, *f_4_-ratio* tests and *f*_d_ statistics [44,47,48] (Table S7).

Comparisons were also made in the 2 Mb region of apomixis loci as defined in *Citrus* [6] and *Fortunella* (see below) (Fig. 2f), with the rest of the genome. To measure introgression, *D* and *f*_d_ statistics were used in 25 kb windows and *D_xy_* was used to measure divergence (Figs S14–S16). Pummelo, mandarin and Hongkong kumquat were used with atalantia as the outgroup to measure introgression between mandarin and Hongkong kumquat. Compared with the rest of the genome introgression level (*D *= 0.4065; *f*_d _= 0.0950), analyses revealed no significant evidence for introgression of the apomixis region (*D =* 0.4353; *f*_d_* = *0.1336*)*.

Introgression is expected to decrease population divergence; therefore, the apomictic region was reanalyzed using the *D_xy_*statistic. However, a similar level of divergence at the apomixis region was observed between the Hongkong kumquat and mandarin groups (*D_xy__ _*= 0.2597) and across the rest of the genome (*D_xy__ _*= 0.2797) (Fig. 2f). Additional comparisons using other sets of populations were similar because they revealed no clear differences at the apomixis region compared to the rest of the genome (Figs S17 and S18). Collectively, these analyses suggest that introgression is not the primary cause of the wide distribution of apomixis across *Citrus* and *Fortunella*.

### Structural and deleterious burden in apomictic and sexual populations

**Figure 3.**
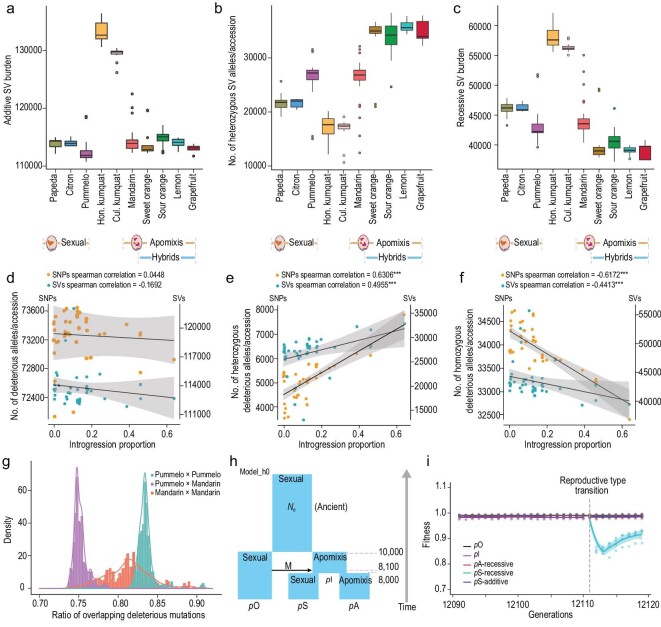
The SVs and deleterious mutations in apomictic and sexual populations. (a) The number of SVs per individual in 10 populations under the additive selection model. (b) The number of heterozygous SV alleles per individual and (c) the number of SVs per individual in 10 populations under the recessive selection model. (d–f) The correlation between the introgressed proportion of the genome and (d) the number of deleterious variants, (e) the number of heterozygous deleterious variants and (f) the number of homozygous deleterious variants, with SNPs and SVs. (g) Distribution of deleterious mutations in hypothetical diploid individuals for three sets of hypothetical crosses. The x axis indicates the ratio of overlapping deleterious mutations in any hypothetical diploid individual, and the y axis indicates the density of the ratio. (h) The demographic model used in forward simulation was inferred from SMC++ analyses. Going forward in time, after a burn-in period of 10^*^N generations (100k generations for model_h0), the ancestral sexual population splits into two subpopulations, one capable of apomixis (*p*I) and the original ancestral population, *p*O. After the split into *p*O and *p*I, a single orientation of introgression was introduced such that 10% of the ancestry of *p*I came from *p*O. Subsequently, the *p*I population split results in *p*S (sexual) and *p*A (apomictic) (reproductive system shifts in the split time). (i) Forward simulations under a model of recessive selection for three demographic scenarios presented in (h) and two reproductive systems with additive and recessive selection. The dashed lines represent the time of the reproductive type transition.

Apomixis in *Citrinae* via nucellar embryony preserves the maternal genotype, which may result in the accumulation of deleterious mutations and SVs in the genome. For these analyses, *Atalantia* and *Poncirus* were used as outgroups to reduce reference bias [49] and SVs and deleterious mutations were investigated after divergence in the 10 populations defined by STRUCTURE. A total of 139 241 reliable SVs were identified from the 234 accessions as described in Methods, 64.2% of which were deletions, 16.6% duplications and 15.5% translocations (Fig. S19). The SV amount varied in different populations under the additive model, and the highest burden was evident in Hongkong kumquat, probably reflecting a decrease in effective population size (Fig. 3a and Fig. S20a). Hybrid populations with apomictic reproduction (e.g. sour orange, sweet orange, lemon and grapefruit) had ∼1.45 times more heterozygous deleterious variants than populations with sexual reproduction (papeda, citron and pummelo) (Fig. 3b and Fig. S8). The recessive model shows a dramatic reduction (11.46% on average) in hybrid apomicts compared to sexually reproducing populations (Fig. 3c). Those SVs are likely to be primarily deleterious and we hypothesize that hybrid apomicts hide the SVs in heterozygous state from recessive selection [40]. Therefore, the hybrids with only sexual reproduction might rapidly expose the recessive deleterious variants. If it is true, this hypothesis explains why the observed natural hybrids derived from the crosses between apomictic and sexually reproducing samples are typically apomictic.

We also predicted genome-wide deleterious mutations using Sorting Intolerant From Tolerant (SIFT). A total of 174 220 deleterious mutations (defined by SIFT score ≤0.05) and 43 470 loss-of-function mutations were predicted. Patterns of the deleterious mutations were consistent with that of SVs, in that there was a lower recessive deleterious burden (7.39%) and more heterozygous SVs (∼2.62 times) in apomictic populations compared to sexually reproductive populations (Fig. S21).

Introgression of foreign DNA can be adaptive and also a means to escape deleterious burden [50]. We therefore studied the association between deleterious burden and the length of introgressed fragments in the genomes of mandarin and pummelo populations. Species-specific markers were used for detecting the proportion of introgression [2,42] (Fig. S22). The introgression proportion positively correlated (Spearman correlation = 0.6306, *P* = 4.4783e-6) with the number of heterozygous deleterious variants, and negatively correlated (Spearman correlation = −0.6172, *P* = 8.1099e-6) with the number of homozygous deleterious variants (Fig. 3d–f). SVs had a similar trend in that the number of heterozygous SVs positively correlated with the length of introgression regions (Spearman correlation = 0.4837, *P* = 6.2498e-4) and the number of homozygous SVs were negatively correlated with the length of introgression regions (Spearman correlation = −0.4533, *P* = 2.7127e-3) (Fig. S21).

To examine the effect of apomixis during citrus breeding on deleterious genetic elements, three hypothetical crosses were used to estimate the ratio of overlapping deleterious variants in hypothetical diploid individuals under the recessive selection [51]. This model included outcrosses within the mandarin group, outcrosses within the pummelo group and an outcross between mandarin and pummelo groups. The analysis was down sampled to eliminate the influence of group size. A 7.2% decrease of the putative ratio of overlapping deleterious mutations was observed in crosses between two groups compared to crosses within the same group (Fig. 3g). These results support the concept that hybrid cultivars derived from mandarin and pummelo have reduced recessive deleterious burden.

Simulations were performed using the demographic model presented in Fig. 3h (model_h0), inferred from the Hongkong kumquat, and introduced introgression events under two reproductive modes (Figs S20 and S23). Under the recessive model, the reproductive mode transition from apomixis to sexual reproduction resulted in a population fitness decline of 13.48 ± 0.87% immediately after the transition but subsequently recovered (Fig. 3i). In contrast, the population that remained apomictic maintained population fitness. This result parallels similar studies of the effects of clonal propagation in grapevines [32], where fitness is higher under clonality because deleterious variants remain hidden in the heterozygous state.

### The same locus contributes to apomixis in both *Fortunella* and *Citrus*

**Figure 4.**
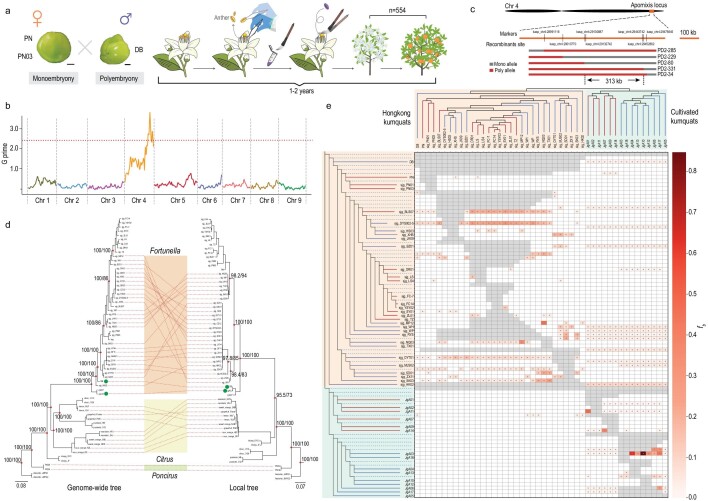
The candidate locus underlying the transition from sexual reproduction to apomixis in *Fortunella*. (a) A diagram of the F1 genetic family. arising from crossing two sexual monoembryonic Hongkong kumquats, ‘PN’ and ‘PN03’, as female parents, with ‘DB’, an apomictic Hongkong kumquat as the male parent. (b) Identification of genomic regions associated with apomixis using bulked segregant analyses. Horizontal dashed lines denote significance levels (Benjamini-Hochberg, *P* < 0.01). (c) Recombination events identified using KASP markers in the ‘PN’ × ‘DB’ population. (d) The discordance between the genome-wide phylogenetic tree and the local phylogenetic tree at the locus underlying the apomixis transition for 63 individuals including Hongkong kumquats and cultivated kumquats (with points). (e) The branch-specific statistic *f_b_* identified an excess sharing of derived alleles between the different branches in *Fortunella*. The asterisk denotes block jackknifing significance (Benjamini-Hochberg, *P* < 0.001). Outgroup, *Atalantia*.

Hongkong kumquats, depending on the accession, can produce seed purely via the sexual process or undergo nucellar embryony where the zygotic embryo may or may not survive (apomixis). To locate the underlying locus of apomixis in *Fortunella*, we constructed a segregating population using the apomictic Hongkong kumquat line ‘DB’ in a cross with two sexually reproducing Hongkong kumquats, ‘PN’ and ‘PN03’, as female parents (Fig. 4a and Fig. S24). A total of 544 offspring (Table S9) were phenotyped as apomictic or sexually reproducing with a ratio of 290:264 (1:1, *P *= 0.2693, chi-squared test). In addition, two bulked F1 populations derived from the sexual ‘PN’  ×  apomictic ‘DB’ cross were sequenced, one containing sexually reproducing progeny and the other containing apomictic progeny—forming polyembryonic seed (Fig. 4b). The differences in SNP indices (ΔSNP index) between the two different bulked samples and the evaluation of G prime value [52] identified a single region on chr4:23.3 Mb–32.8 Mb (*P* < 0.01, FDR) (Fig. 4b and Fig. S25). Furthermore, kompetitive allele specific PCR (KASP) markers were developed to further narrow the locus to a 313 kb region (Fig. 4c). One highly divergent SNP was found that clearly distinguished sexual and apomictic progeny in the 554 offspring of the sexual ‘PN’  ×  apomictic ‘DB’ cross (Fig. S26).

A phylogeny based on the 313 kb region was built, and it delimitated apomicts and sexual reproducers in both *Fortunella* and *Citrus* (Fig. 4d). We compared the discordance between the local tree and genome-wide phylogenetic tree and noticed intersections among operational taxonomic units (OTUs) within cultivated kumquat and wild Hongkong kumquat (Fig. 4d). For example, the apomixis region in Hongkong kumquat was closely related to cultivated kumquat. To clarify the effects of introgression on the evolution of apomixis in wild Hongkong kumquat and the cultivated kumquat, we examined the genome-wide *f_b_* statistics in all samples of *Fortunella*. There were nine individuals with significant *f_b_* values (Fig. 4e), and the varieties ‘DB’ (*f_b_* = 0.1059 ± 0.0049) and ‘HK02’ (*f_b_* = 0.1275 ± 0.0052) showed the highest *f_b_* statistic. Together with the *D* statistic (0.1832 ± 0.0052 and 0.1971 ± 0.0054 for ‘DB’ and ‘HK02’ respectively) and the *f_4_-ratio* (0.1435 ± 0.0071 and 0.1673 ± 0.0079 for ‘DB’ and ‘HK02’ respectively) in ‘DB’ and ‘HK02’ (also indicated in Fig. 4d), these results suggested that apomixis of at least some samples in *Fortunella* originated via introgression (Table S10).

**Figure 5.**
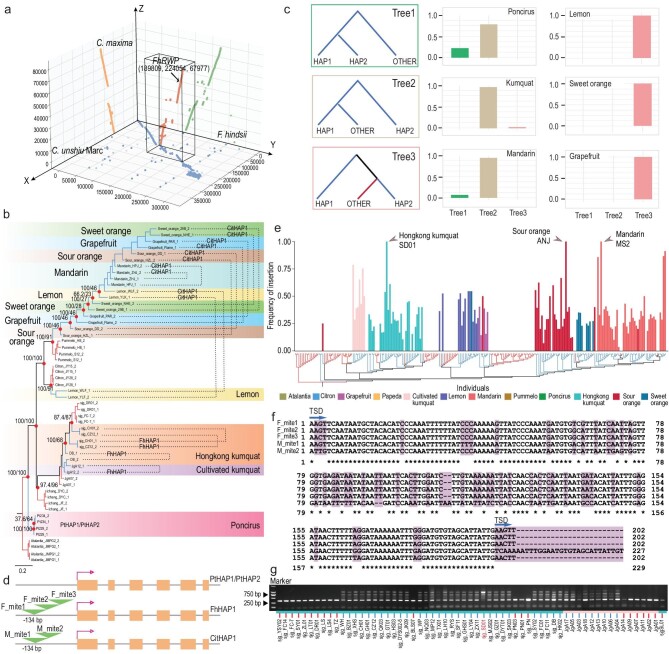
Heterozygous MITE insertions in *Fortunella* and *Citrus.* (a) Conserved region in the apomixis candidate locus from three published F1 genetic populations in *Fortunella* and *Citrus*. The alignments of the located apomixis region are derived from mandarin (*C. unshiu* Marc.), pummelo (*C. maxima*) and Hongkong kumquat (*F. hindsii*). The x axis represents the previously identified 380 kb apomixis region of the mandarin genome, the y axis represents the 313 kb apomixis region of the Hongkong kumquat genome and the z axis represents the 80 kb apomixis region of the pummelo genome. (b) A haplotype phylogenetic tree of the apomixis region. Apomictic individuals are highlighted using colored bands, and the dotted line links two haplotypes from the same individual. (c) Comprehensive topologies at the apomixis region of the haplotypes in apomictic samples identified haplotype divergence in apomictic individuals (left). HAP1 presents one haplotype with the MITE insertion in apomictic *Fortunella* and *Citrus* (not evident in *Poncirus*). Another haplotype is denoted as HAP2. OTHER represents a haplotype from other samples. The haplotypes from sexual and apomictic varieties are represented by different colors. Bar plots denote the frequency of each topology in six apomictic groups (right). (d) Diagram showing the MITE insertions in *Fortunella* (Fh) and *Citrus* (Cit). No insertions are evident in the *Poncirus PtRWP* gene promoter. Three MITE insertions are found in the *FhRWP* (*Fortunella*) promoter and the two MITE insertions are evident in the *CitRWP* promoter of *Citrus* varieties. (e) The frequency of MITE insertions in 232 individuals, excluding two *Poncirus* samples with low reads coverage. The phylogenetic tree was constructed using the whole-genome SNP data set. Sexual reproduction and the capacity for apomixis are represented by different colors. (f) Alignment of the repetitive MITE insertions in *Fortunella* and *Citrus*. Asterisks indicate identical bases, while differences are highlighted. (g) Verification of MITE insertions in 61 individuals of *Fortunella*. The different reproductive types are represented by different colors. Of the 40 samples, 39 contained heterozygous MITE insertions, and one plant, ‘SD01’, containing homozygous MITE insertions is highlighted.

We compared the apomixis region identified from the *Fortunella* F1 population with two other published genetic populations in *Citrus* to examine genic signatures. These included an F1 population derived from ‘Kiyomi’ [‘Miyagawa wase’ (*C. unshiu Marc.*) × ‘Trovita’ orange (*C. sinensis* (*L*.) *Osbeck*)] × ‘Miyagawa wase’ (*C. unshiu Marc.*) [34] and an F1 population derived from ‘Fairchild’ [‘Clementine’ (*C. clementina*) × ‘Orlando’ (*Citrus × tangelo*)] × ‘HB’ (*C. maxima*) [6]. The sequence's alignment was performed to further define the major cause associated with the initiation and development of nucellar embryos in apomixis genomic regions shared between Hongkong kumquat (313 kb), mandarin (380 kb) and pummelo (80 kb) genomes (Fig. 5a). The minimum overlap among these three regions contained the *FhRWP* gene, an RWP-RK domain transcription factor that is homologous to the *CitRWP* gene reported to contribute to apomixis in mandarin [6,36].

### Heterozygous MITE insertions underlying apomixis in *Citrinae*

To study the patterns of genetic variation in the apomixis determination region across *Citrinae*, a phylogenetic tree of the 313 kb region (*F. hindsii* genome) was constructed based on haplotypes (Fig. 5b and Fig. S27). The results suggested multiple appearances of apomixis in *Citrinae*. Moreover, two haplotypes of the same individual were not always clustered in the same branch or clade, which suggests different origins of apomixis in different populations. In general, three typical patterns (tree1, tree2 and tree3 in Fig. 5c) were observed in apomictic individuals (Fig. 5c).

Previous studies indicated that a MITE insertion in the promoter of a *CitRWP* gene is associated with 213 apomictic *Citrus* accessions [6]. We combined polymerase chain reaction (PCR) and circular consensus sequencing (CCS) to clarify the complex structure in the *FhRWP* promoter, including 103 samples in *Citrinae* (Table S13). The results confirmed that a MITE insertion was strongly associated with apomixis and apomictic haplotypes, except within *Poncirus* and its related individuals (Table S13). The haplotypes in sexually reproductive individuals did not contain the MITE insertion. Furthermore, MITE insertions in *Fortunella* and *Citrus* were distinguished by different copy numbers (2 or 3) of the MITE (Fig. 5d and f). This contrasts with our prior results that detected only one MITE insertion of 203 bp, based on Illumina short read sequencing and/or Sanger sequencing in *Citrus* [6,36,37]. Two apomictic haplotypes with MITE insertions in the *RWP* promoter were observed in *Fortunella* and *Citrus.* One haplotype was 596 bp in *Fortunella* containing three similar MITE sequences of 202 bp repeats, including two overlapping 5 bp tandem site duplication (TSD) sequences. Another haplotype, the MITE insertion in *Citrus*, was 424 bp characterized by two different MITE insertions of ∼202 bp and ∼227 bp, which contained overlapping 5 bp TSD sequences (Fig. 5f and Table S13). The differing number of MITEs and their different sequences suggest the possibility that apomixis alleles originated on separate occasions.

To further analyze the variability of the MITE insertion in *Citrinae*, we investigated the frequency of MITE insertions in the aforementioned population using Illumina paired-end (PE) read resequencing. The MITE insertions were not observed in the promoters of purely sexually reproducing varieties (Fig. S28). Among the apomictic individuals analyzed in *Fortunella* and *Citrus*, 97.6% of accessions (124 out of 127) contained only one of the above-mentioned apomictic haplotypes and were heterozygous for the MITE insertion in the *RWP* gene promoter (Fig. 5e). Hongkong kumquat accession ‘SD01’, mandarin variety ‘MS2’ and sour orange variety ‘ANJ’ were homozygous for the MITE insertion in their *RWP* promoter. Phylogenetic analyses of the 313 kb apomixis region in the above three varieties showed that the two observed haplotypes of MITE insertions in the *RWP* gene promoter came from different clades (Fig. S29). The observed pattern of MITE insertion was also verified using agarose gel electrophoresis in 61 *Fortunella* individuals, including the individual ‘SD01’ (Fig. 5g). Collectively, these findings revealed that MITE insertions are very common in the heterozygous state, which may be related to haplotype differences in apomictic varieties.

### Identification of factors that interact with the MITE element in *FhRWP*

**Figure 6.**
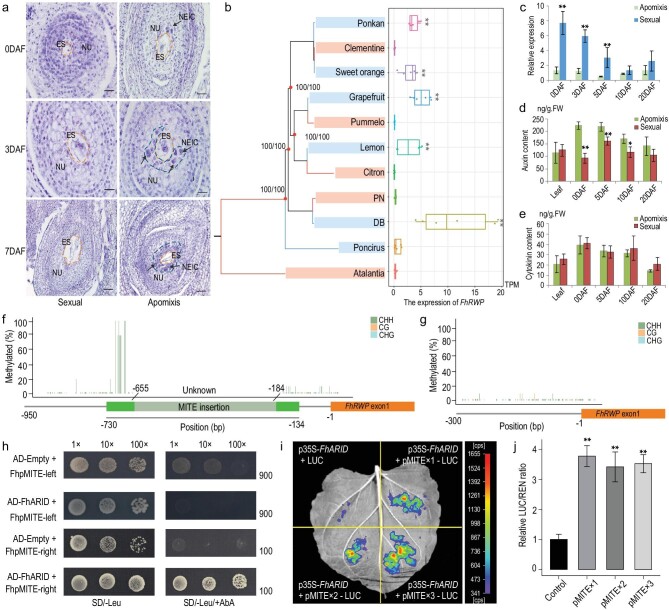
Molecular characterization of the regulation of apomixis in *Fortunella* and *Citrus*. (a) Cytological comparisons of three developmental stages of ovules. 0, 3 and 7 days were defined as flowering and post-flowering stages. The pictures show nucellar embryo initiation in apomictic Hongkong kumquat compared with the absence of nucellar embryo initials during sexual events in a monoembryonic variety at 40 × magnification. NU, nucellar tissue. ES, embryo sac. NEIC, nucellar embryo initiation cell. (b) The expression of *FhRWP* was calculated from 11 independent ovule RNA-seq samples using a transcripts per million (TPM) value. The phylogenetic tree (1000 bootstrap and SH-like approximate likelihood ratio) was constructed using the whole-genome SNP data set. Different colored branches represent individuals undergoing different reproductive types. The *FhRWP* was expressed in apomictic *Fortunella* and *Citrus* samples, but not expressed in sexually reproducing samples within the same clade and the *Poncirus* sample (^**^P value < 0.01, two-tailed Student's t-test). (c) RT-qPCR verified the expression of *FhRWP* in Hongkong kumquat at five development stages. Statistically significant levels of expression are marked as ^*^P value < 0.05, ^**^P value < 0.01 in (c–e), two-tailed Student's t-test. (d and e) Comparison of content of auxin and cytokinin in ovules of sexually reproductive and apomictic Hongkong kumquat. (f and g) Estimation of the cytosine methylation levels of the *FhRWP* promoter using bisulfite sequencing (BS) PCR in young leaves from (f) apomictic and (g) sexually reproductive Hongkong kumquats. Three independent experiments were undertaken with similar results. (h) Yeast one-hybrid assays identified interactions of the AT-rich specific DNA-binding protein *FhARID* with the MITE insertion in the *RWP* promoter region. FhpMITE-left denotes the left part (119 bp) of the one MITE insertion in Hongkong kumquat, while FhpMITE-right denotes the right part (91 bp). The overlap of both fragments was 8-bp. (i) Transient promoter activity assays were carried out using three different copies of FhpMITE-right (short in pMITE) driving the luciferase (LUC) reporter gene, along with the *FhARID* gene and the empty vector as an internal control. REN, renilla luciferase, an internal control. (j) Relative LUC/REN ratio values indicate mean ± s.d. (*n*  =  6 reactions). ^**^P value < 0.01 (two-tailed Student's t-test).

To identify the molecular basis of apomixis in *Fortunella* and *Citrus*, we began by cytologically analyzing the growth of the nucellar embryo initials that were evident at anthesis in ovules of apomictic Hongkong kumquat but not in sexual Hongkong kumquat (Fig. 6a). We compared the expression of *FhRWP* in six apomictic varieties/accessions (*Poncirus*, Hongkong kumquat ‘DB’, lemon, grapefruit, sweet orange and ponkan) and five sexually reproducing ones (*Atalantia*, Hongkong kumquat ‘PN’, citron, pummelo and clementine) over two periods, including 7 days before flowering and 7 days after flowering (Figs S30 and S31 and Table S11), by using transcriptome analysis (Figs S30–S32 and Table S11). *FhRWP* expression was detected in the apomictic haplotypes with MITE insertions over both periods but not in the sexually reproducing accessions without MITE insertions (Fig. 6b). We confirmed this result with reverse transcription quantitative real-time PCR (RT-qPCR) over five developmental stages, 0, 3, 5, 10 and 20 days after flowering (Fig. 6c). The growth of nucellar embryos strongly correlated with higher levels of auxin before day 10 (*P* < 0.05, two-tailed Student's t-test) (Fig. 6d and e and Fig. S33).

To study the factors involved in the regulation of *FhRWP* gene expression, we evaluated the pattern of methylation on the MITE elements within the promotor of young leaves, because *FhRWP* is expressed in the young leaves of apomictic Hongkong kumquat, but not in sexual Hongkong kumquat (Fig. S34b). CHH, CG and CHG methylation levels were similar in *FhRWP* promoter sequences in the genomic DNA of the leaves of apomictic and sexual samples. However, the apomict sequence contained 10 sites with CHH methylation at the upstream end of the inserted MITE sequences (Fig. 6f and g). Therefore, it is unlikely that methylation levels as examined here are preventing expression of the gene in the leaves of sexual accessions. It is not possible to exclude the possibility that this may be the case in the nucellus of the ovule.

Yeast one-hybrid (Y1H) analyses were used to better understand the factors interacting with the MITE-containing *FhRWP* promoter in apomictic Hongkong kumquat that facilitates ovule gene expression. We divided the 202 bp MITE (FhpMITE) sequence into two parts (FhpMITE-left and FhpMITE-right, details in Methods) and separately performed Y1H screens to maximize the exposure of interactors to the complex secondary structure of the MITE sequences. We identified a transcription factor, *FhARID* (Fh3g37720) (Fig. S35), located on a different chromosome to the apomictic region, that encoded an AT-rich interaction domain-containing protein, which bound to the FhpMITE-right fragment (short in pMITE, see Methods for this 91 bp sequence) (Fig. 6h). *FhARID* was specifically expressed in apomictic Hongkong kumquat ovules (Fig. S35b). Two homologs of *FhARID, FhARID2* (Fh9g13670) and *FhARID3* (Fh4g25380), in the Hongkong kumquat genome, did not have an activation function on *FhRWP* (Fig. S35a).

To investigate if the number of MITE sequences was important for FhARID-induced activation of *FhRWP* expression, we artificially constructed promoters with one, two and three copies of pMITE sequences and conducted a dual luciferase assay via transiently transformed tobacco (Fig. 6i). The results showed that *FhARID* binds the pMITE sequences and activates linked reporter gene expression (^**^P < 0.01, two-tailed Student's t-test), but the number of pMITE sequences did not show significant differences in *FhARID*-mediated activation (two copies, *P* = 0.4271; three copies, *P* = 0.3064, two-tailed Student's t-test) (Fig. 6j). However, the direct influence of multiple MITEs in the promoter of the *RWP* on gene expression and nucellar embryo initial cell induction in the ovule remains to be determined.

## DISCUSSION

### Induction of nucellar embryony in *Citrus* and *Fortunella*

Our exploration of the molecular basis of apomixis in *F. hindsii* used a segregating population to identify the major locus that contributes to nucellar embryony. The locus encompasses 313 kb, and it includes the *FhRWP* gene, which is orthologous to the *CitRWP* gene that has been hypothesized to induce apomixis in *Citrus*. Analysis of *FhRWP* and its homologs across *Citrinae* revealed a striking pattern of MITE insertions in the 5’ promoter regions of these genes. Apomictic accessions in *Citrus* and *Fortunella* contain two or three MITE insertions in the *RWP* promoter, respectively, while sexually reproducing *Citrinae* individuals lack MITE insertion [6,53]. These MITE insertions correlate with *RWP* gene expression levels in the nucellus of apomicts [36] and act as apparent binding targets of the transcriptional regulator *FhARID*, which may mediate gene expression and embryogenic induction. An *Arabidopsis* gene, *AHL15*, has been reported to have a similar binding pattern as *FhARID*; it harbors an AT-hook motif and mediates somatic embryogenesis [54]. Elucidating the contribution of *FhARID* to apomixis will require *FhARID* knockouts in apomictic Hongkong kumquat, which has a short juvenile phase facilitating functional characterization [8,14].

### Evolution of apomixis loci in *Citrinae*

There appears to be heterogeneity in genes causing nucellar embryony in *Citrinae*. Apomictic *Poncirus* varieties do not have MITE insertions in the 5^′^ promoter of the *RWP* gene and the gene is not expressed in nucellar ovule cells, suggesting another causal gene [55]. The pattern of allelic heterogeneity, with different numbers of MITE insertions and with distinct sequences in *Citrus* and *Fortunella*, suggests the possibility that apomixis arose more than once among *Citrinae*. Phylogenetic analysis of both whole-genome data (Fig. 2a) and haplotypes in the apomixis region (Fig. 5b) supports the idea of the frequent appearance of the trait across different lineages.

An alternative explanation for the phylogenetic distribution of apomixis across *Citrinae* is introgression [29]. Introgression has been an important force in the evolution of citrus. A recent study identifying a sexually reproducing wild citrus species suggested that hybridization contributed to the transition from sexual to apomixis in mandarins [37]. Previous work has shown that mandarins contributed to modern cultivars through hybridization with pummelo and citron [4,55]; and here we have uncovered additional haplotype evidence for the transmission of the apomictic phenotype through hybridization in *Citrus* and *Fortunella* (Fig. 5b).

Collectively, our analyses support prior work that suggests that hybridization in *Citrinae* is commonly associated with the domestication of citrus cultivars (Fig. 2a and Table S7). Introgression may have been common because these genera have incomplete reproductive isolation, perhaps due in part to a longer generation period than annuals [56]. For example, artificial crosses between trifoliate orange (*Poncirus*) and pummelo have been successful [57]. Crosses between trifoliate orange and mandarin also have a high rate of success (up to 51.3%), however, embryo rescue is required [58].

Signals of introgression among different genera were not detected at the known *RWP*-containing apomixis locus (Figs. 2f, 4d and 5b). The lack of clear introgression signals further suggests that parallel evolution has driven the evolution of apomixis in *Fortunella* and *Citrus.* Parthenogenesis in Asteraceae in *Taraxacum* and *Pilosella* genera is a clear case of parallel evolution [27,59]. MITE insertions in the promoter regions of the same gene appear to be causing the misexpression of an embryo-inducing gene in the egg cell in the absence of fertilization [27]. This appears to be the case in some *Citrinae* varieties as the same *RWP* gene is recruited and its expression profile appears changed by transposon insertion to enable ectopic embryos to form. The massive nucellus of the citrus ovule appears to provide sufficient nutrients for early growth. Developmentally there must be spatial and temporal factors restricting embryogenesis in the nucellus as not all cells undergo embryogenesis. Typically, a single embryo forms in a seed via the sexual process. Support of multiple embryos requires access to nutrients, and it is unclear what pathways are recruited for this, and for access to post-fertilization endosperm.

### Implications of apomixis in citrus breeding

The genomic patterns observed during the course of these analyses have shown that apomictic citrus individuals accumulate heterozygous deleterious mutations in the genome via hybridization and introgression (Fig. 3b and e), including hemizygous genes, at a higher level than sexually reproducing taxa. Thus, the apomictic reproductive mode has a detectable effect on deleterious load because apomictic populations are likely to accumulate more heterozygous deleterious mutations with commensurately lower levels of recessive deleterious burden (Fig. 3c and f).

It seems to be that those modern apomictic citrus cultivars can hide deleterious variants in a heterozygous state. Therefore, population fitness may be maintained, assuming the deleterious variants are hidden in a heterozygous state [60]. The flip side to this argument is that the heterozygous burden is eventually exposed in citrus cross-breeding and results in a resemblance to inbreeding depression [51]. The transition from apomixis (i.e. loss of apomixis) to complete sexual reproduction could potentially result in segregation distortion due to expression of recessive lethal alleles [24].

Clearly the production of clonal maternal embryos via the process of facultative apomixis in citrus is not identical to clonal propagation via vegetative cuttings. Facultative apomixis in citrus maintains low levels of functional sexual seed formation [15]. Theoretical evidence indicates that residual sex matters for facultative apomixis. Because sexual reproduction can expose heterozygous deleterious mutations that can halt the Muller's ratchet. Muller's ratchet, which is the accumulation of deleterious mutations [33]. Population fitness will be dependent on how many generations of asexual reproduction have taken place and the frequency of sexuality [33,61].

The forward simulations undertaken in this study under a demographic model for citrus suggest that populations with <100 generations of asexual reproduction can recover their fitness within ∼10 generations of sexuality, but populations with >500 generations of asexual reproduction exhibit reduced population fitness, by an average of 7.8%, after the population eventually reaches equilibrium (Fig. S23). Similarly, grafting, commonly used in citrus propagation, will hide heterozygous deleterious alleles from recessive selection [62]. We propose that citrus breeding should focus on wild and semi-wild accessions, which limits the heterozygous deleterious burden by ensuring recent sexual reproduction. An ongoing challenge is to take advantage of genomic data to predict the deleterious burden in advance when selecting parents for breeding, providing a potentially powerful method of improving breeding in clonal crops [63].

## MATERIALS AND METHODS

See Supplementary Data.

## DATA AVAILABILITY

Data supporting the findings of this work are available within the paper and its Supplementary Data files. Genome sequences and sequencing data are accessible through NCBI under the BioProject ID PRJNA735863. SV and SNP VCFs, the TE insertion modified genome, expression data set, genome assembly and annotation of genes and transposable elements are available at https://zenodo.org/record/5748662. Custom scripts and workflows are available at https://github.com/wangnan9394/apomixis_parallel_evolution.

## Supplementary Material

nwac114_Supplemental_Files
